# Theoretical and practical training improves knowledge of the examination guidelines of the International Standards for Neurological Classification of Spinal Cord Injury

**DOI:** 10.1038/s41393-020-00578-1

**Published:** 2020-11-17

**Authors:** Steffen Franz, Laura Heutehaus, Sina Weinand, Norbert Weidner, Rüdiger Rupp, Christian Schuld

**Affiliations:** Spinal Cord Injury Center, Heidelberg University Hospital, Schlierbacher Landstraße 200a, 69118 Heidelberg, Germany

**Keywords:** Physical examination, Outcomes research, Spinal cord diseases

## Abstract

**Study design:**

Prospective pre–post study.

**Objectives:**

International Standards for Neurological Classification of Spinal Cord Injury (ISNCSCI) represents the most frequently used assessment to determine the level and severity of a spinal cord injury (SCI). The guidelines for ISNCSCI are complex and challenging. Knowledge of its correct execution needs to be imparted precisely. The aim of this study was to investigate whether hands-on instructional courses can increase the knowledge of the ISNCSCI examination guidelines.

**Setting:**

European Multicenter Study about SCI.

**Methods:**

Before and after the instructional courses, participants were asked to complete questionnaires. The set of questions covered the most important aspects of the examination guidelines. Attendees were asked to self-rate their occupation and experience in ISNCSCI.

**Results:**

The comparison of pretest and posttest results of 164 attendees from 2014 to 2018 revealed an improvement of knowledge reflected by an increase of correct answers from 66 ± 17% before to 89 ± 11% after the course (*p* < 0.01). The improvement was not associated with occupation (*p* > 0.1). However, the correctness of pretest results differed concerning both the period of experience with ISNCSCI (*p* < 0.05) and the course language (*p* < 0.01), while the frequency of execution resulted in differences in the posttest (*p* = 0.01).

**Conclusions:**

Instructional courses substantially improve knowledge of the ISNCSCI examination guidelines. Differences in knowledge present before the course leveled off after the course. Comprehensive theoretical training is strongly recommended to ensure reliability and validity of ISNCSCI examinations in clinical routine and research. Albeit being practiced in the instructional courses, the benefit of hands-on training still needs to be systematically evaluated in future studies.

## Introduction

Since its introduction in 1982, the International Standards for Neurological Classification of Spinal Cord Injury (ISNCSCI) evolved to the key instrument with regard to the clinical characterization of spinal cord injury (SCI) [1, 2]. Thus, the ISNCSCI and its classification parameters gained relevance in clinical routine as well as in clinical trials [3, 4]. In the scientific context, ISNCSCI is used not only as an outcome measure, but also to define inclusion and exclusion criteria and to facilitate stratification and sub-grouping for analysis of data. For instance, the accurate and comprehensive collection of reliable and valid data within the European Multicenter Study about Spinal Cord Injury (EMSCI) led to important findings, such as prediction rules for estimation of the probability of ambulation [5] and for urinary continence 1 year after injury [6] based on an ISNCSCI examination within 4 weeks after injury. From a clinical perspective, ISNCSCI upper and lower extremity motor scores help to identify the most suitable rehabilitative strategies, in terms of compensatory and restorative treatment tracks. To ensure a correct determination of all ISNCSCI variables, two essential skill sets are required: (1) a reliable and valid clinical examination and (2) an accurate classification of the examination results. Referring to the latter, several articles have been published, showing the relevance of standardized training to achieve high classification accuracy [7–9]. In contrast, evidence on the importance of attaining detailed knowledge of the challenges and requirements of an accurate ISNCSCI examination is still sparse and less comprehensive [10]. This investigation aimed to systematically evaluate the immediate effects of imparting the ISNCSCI examination guidelines by means of a ten-item questionnaire in the setting of ISNCSCI instructional courses held within the EMSCI network.

## Methods

This study was conducted within the framework of EMSCI (https://emsci.org), approved by the ethics committee of the Heidelberg University (S-188/2003). To ensure high data quality, ISNCSCI instructional courses including success monitoring (pre- and posttests including posttest-only questions) are held on a regular basis as part of the EMSCI quality management system according to ISO 9001:2015.

### Concept of the ISNCSCI instructional course

The instructional course was designed to be held over 1.5 days, but could also be shortened to a single day, if necessary. It was offered alternately in German and English language. The ISNCSCI sensory and motor examinations are in the focus of the first half-day. This part of the course covers all theoretical aspects and practical clinical challenges of the exam. A professionally supervised hands-on assessment of in-patients is conducted after the theoretical part (half-day). The last part of the course is then focused on ISNCSCI scoring, scaling, and classification. It comprises a theoretical part, which is followed by an interactive classification of both the previously assessed in-patients and a collection of difficult cases [11].

### Success monitoring and its development

As part of the EMSCI quality management system, two questionnaires have to be completed at the beginning and after completion of the course (pre- and post-course test). The questionnaires were offered in the course language German or English, respectively. Initially, all attendees have to do a self-rating regarding their individual characteristics like the occupation group, the general experience with SCI, the experience with the ISNCSCI examination, as well as with the scoring, scaling, and classification part of ISNCSCI. Finally, the frequency of performing hands-on ISNCSCI examinations in clinical routine was assessed [9]. While the actual pretest questionnaire consists of five questions, the course participants have to answer nine questions in the posttest. The first five questions in the pretest are repeated in the posttest (Supplementary Material 1). All questions cover challenging aspects of the clinical examination. The designated question “Q6” was removed from the questionnaire without replacement after the first course, because it was answered 100% correctly.

The questionnaires were developed by an interdisciplinary team of SCI clinicians and researchers, who also are experienced ISNCSCI examiners. All were course instructors with different professional backgrounds, including two physical therapists, one physician, one medical-technical assistant, one computer scientist for medical informatics, and one rehabilitation engineer. The questionnaires were intended to comprehensively cover clinically challenging aspects of the ISNCSCI examination. The most crucial issues of the sensory and the motor examination were defined by means of a consensus process among the members of the interdisciplinary team and identified based on the experience with more than 20 ISNCSCI instructional courses held at the Spinal Cord Injury Center of Heidelberg University, two to three times a year, from 2006 through 2014. The following five steps were considered to be particularly challenging: (1) proper positioning of the patient, (2) accurate application and grading of sensory testing for both light touch and pinprick appreciation, (3) assessment of sacral sparing, (4) evaluation of key muscle function, including the grade dependent positioning, and (5) identification/avoiding of trick movements during muscle testing [12, 13].

The questionnaire was composed of five multiple choice (one key item and four distractors) and of four multiple response questions (four items of which one to five could be keyed as correct, also known as Kprime; Table 1) [14, 15]. Of these, five questions (questions 1–5 [Q1–Q5]) were stated in both pre- and post-course tests and four (Q7–Q10) were solely put in the post-course test. The rationale behind integrating Q7–Q10 into the posttest as explicitly difficult questions was to increase the discriminability between good and excellent attendees. The content of Q7–Q10 was defined in a way that novices, who form the majority of participants in instructional courses [9], could hardly be expected to have a priori knowledge of the correct responses. Based on an interim analysis after four instructional courses, two of the questions were substantially modified (Table 1). The pre- and post-course tests were done as individual tests. Teamwork was discouraged and prevented by the instructors.

**Table 1:** Listing of all tested learning contents arranged according to question number, type, and mode of query.

Question	Type	Mode	Wording	Correct keys
Q1	MC	Pre/Post	A patient reports a strong tingling sensation when being tested with both ends of the safety pin in the pinprick examination. Due to this sensation the patient cannot reliably distinguish between the sharp and dull end of the safety pin. What is the correct grading?	1
Q2	MC	Pre/Post	The patient reports the same tingling sensation when being touched with the cotton tip in the light touch examination. What is the correct grading?	1
Q3	MC	Pre/Post	Which tool does the ASIA define for testing pinprick discrimination?	1
Q4	MC	Pre/Post	What is the meaning of grade 3 in the motor examination?	1
Q5	MR/Kpr	Pre/Post	In which position has the patient to be for the ISNCSCI examination?	2
Q6	MC	Pre/Post	How many key muscles are tested per body side in ISNCSCI’s motor examination?	1
Q7A/B	MR/Kpr	Post	While testing voluntary anal contraction according to ISNCSCI, you, as the examiner, feel a contraction. Which of the following procedures can help in distinguishing a voluntary from a reflex anal contraction?	Modified keys 2
Q8	MR/Kpr	Post	ISNCSCI also contains an examination for deep anal pressure to evaluate the sensory fibers of the spinal segments S4–5. How is it tested?	2
Q9A	MR/Kpr	Post	The C6 key muscle examination (M. ext. carpi radialis) for grades 4 and 5 requires the examiner to put resistance against the patient’s movement. To which direction do you as the examiner put the resistance?	2
Q9B	MC	Post	The C6 key muscle examination (M. ext. carpi radialis) for grades 4 and 5 requires the examiner to put resistance against the patient’s movement. To which direction do you as the examiner put the resistance?	1
Q10	MC	Post	A common compensatory movement during the S1 (plantar flexion) in the grade 3 position is the following active movement?	1

Correct keys indicate the number of correct answers per question. The wording before (A) and after (B) their modification is additionally listed for Q7 and Q9. Q7B is characterized by an unchanged wording of the question, but a modified wording of the given keys. The complete questionnaires, including all keys and distractors, are enclosed as Supplementary Material 1.

*MC* multiple choice, *MR/Kpr* multiple response/Kprime, *Pre* pre-course, *Post* post-course, *Q* question.

### Statistical analysis

As the first step in analysis, the percentage of correctly answered questions per attendee for the pre- and post-course tests was calculated. Main outcome was the improvement between pre- and posttest. Additionally, a successful outcome was assumed if attendees achieved a level of correctness ≥ 90% after the course in a pre–posttest approach. This is based on previous experiences with the evaluation of ISNCSCI classification skills [9]. Due to the expected non-normal distribution of the outcome, nonparametric statistics (Kruskal–Wallis one-way analysis of variance with Dunn’s test of multiple comparisons using rank sums as post-hoc procedure) were used. The difference between pre- and post-course test per question was analyzed with the McNemar’s test. All statistics were done in R [16]. A *p* value below 0.05 was considered statistically significant. Given low sample sizes in certain subgroups of the individual/personal characteristics, some subgroups had to be pooled (Supplementary Material 2): “physical and occupational therapists” were grouped as “therapists” and “other rehabilitation professionals” were grouped with “nurses” as “other”; experiences of “6–10 years” were grouped together with “more than 10 years” as “more than 5 years”; “experts,” “highly experienced,” and “experienced” attendees in both ISNCSCI examination and classification were grouped as “experienced”; frequency of “once a day,” “twice a week,” and “once a week” were grouped as “≥once a week.”

## Results

One hundred sixty-four attendees were trained in 13 ISNCSCI instructional courses from November 2014 to November 2018. Nine of these were held in Heidelberg (Germany) as 1.5-day courses, another three in Murnau (Germany), Salzburg (Austria), and Glasgow (United Kingdom) as 1-day courses.

The training team remained unchanged throughout the study period including the courses outside of Heidelberg. The sensory examination was mostly taught by author SF (12 times) or author LH (once). The motor examination was taught by LH (eight times) or another physiotherapist (five times). The classification part was taught by CS (11 times) or RR (once). Six of the courses were taught in German and seven in English. Most of the attendees were physicians (55%), had <1-year experience in the field of SCI (35%), were novices in ISNCSCI examination (66%) and classification (70%), and did not regularly perform ISNCSCI examinations (45%). A detailed characterization of the participants is shown in Supplementary Material 2.

### Primary analysis

**Fig. 1:**
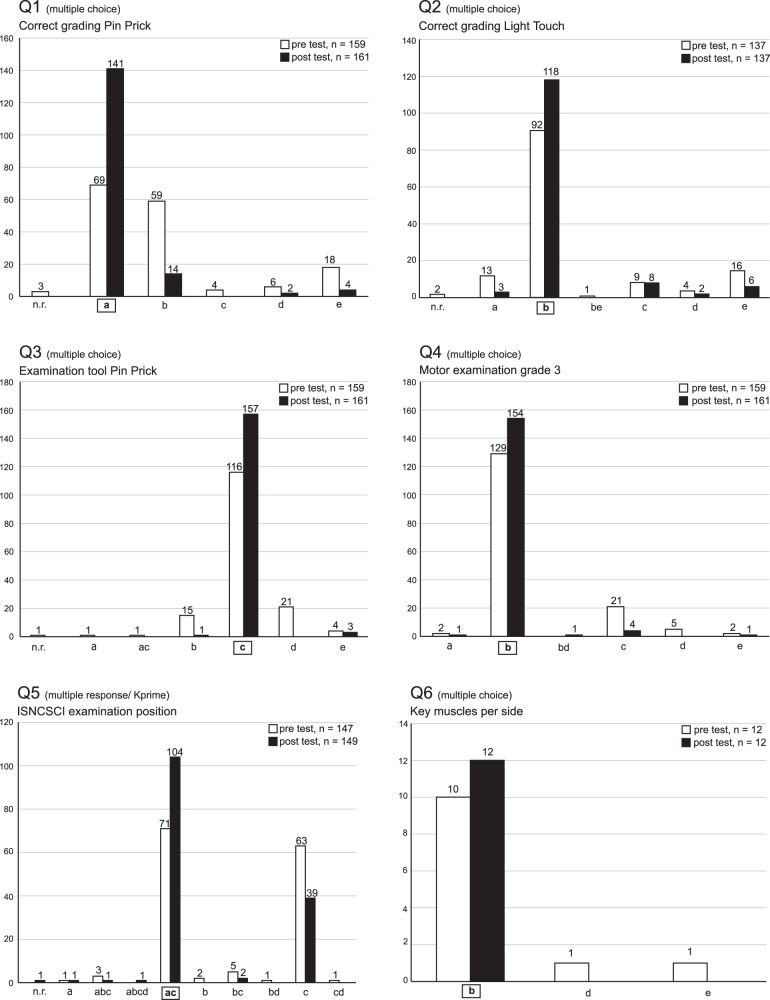
Comparison between pre-course and post-course test results of questions 1–6 (Q1–Q6). X-coordinates denote all selected keys (correct answers) and distractors (wrong answers). Keys are highlighted by a surrounding rectangular frame. Y-coordinates and the numbers above the columns reflect the count of selected distractors and keys. Question type “multiple response/Kprime” allows more than one possible key, while question type “multiple choice” is characterized by only one key and four distractors. In all questions, the correct keys and combination of keys were the most frequently chosen answers. All questions and their contents are completely listed in Table 1. Note that Q6 has been removed after the first course. n.r. no response.

**Fig. 2:**
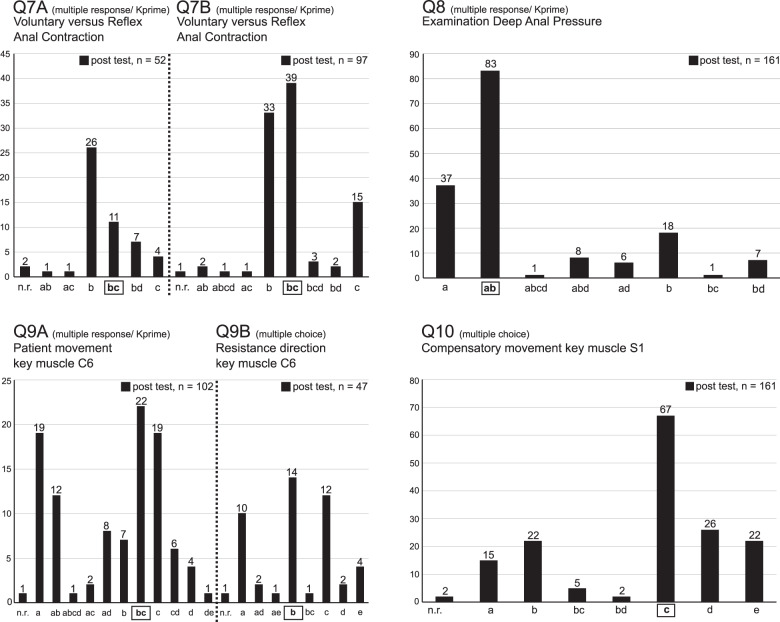
Illustration of results related to the questions that were only part of the post-course test (Q7–Q10). X-coordinates denote all selected keys (correct answers) and distractors (wrong answers). Keys are marked by a rectangular frame. Y-coordinates and the numbers above the columns reflect the count of selected distractors and keys. Question type “multiple response/Kprime” allows in these particular cases two correct keys, while question type “multiple choice” is characterized by only one key and four distractors. Subfigures Q7 and Q9 illustrate both the results of the questions before (Q7A/Q9A) and after (Q7B/Q9B) their modification. Except for the unmodified question Q7A, all other questions were correctly answered in most instances. After modification, attendees have most frequently chosen the correct answers for Q7B and Q9B. Both the unmodified and the modified questions are listed in detail in Table 1. n.r. no response.

In Q1–Q5 (Fig. 1), which were included in both the pre-course test and the post-course test, the mean percentage of correct answers (±SD) significantly increased from pre- to post-course test from 66 ± 17% to 89 ± 11% (*p* < 0.01). More than half of the attendees (56%, *N* = 87) achieved an error-free result in the posttest with 100% correct answers. In Q7–Q10, which were only posed in the posttest, the mean percentage of correct answers was 36 ± 14% (Fig. 2). Posttest-only results of Q1–Q5 (89 ± 11%) versus Q7–Q10 (36 ± 14%) differed significantly (*p* < 0.01).

### Attendee-related analysis

**Fig. 3:**
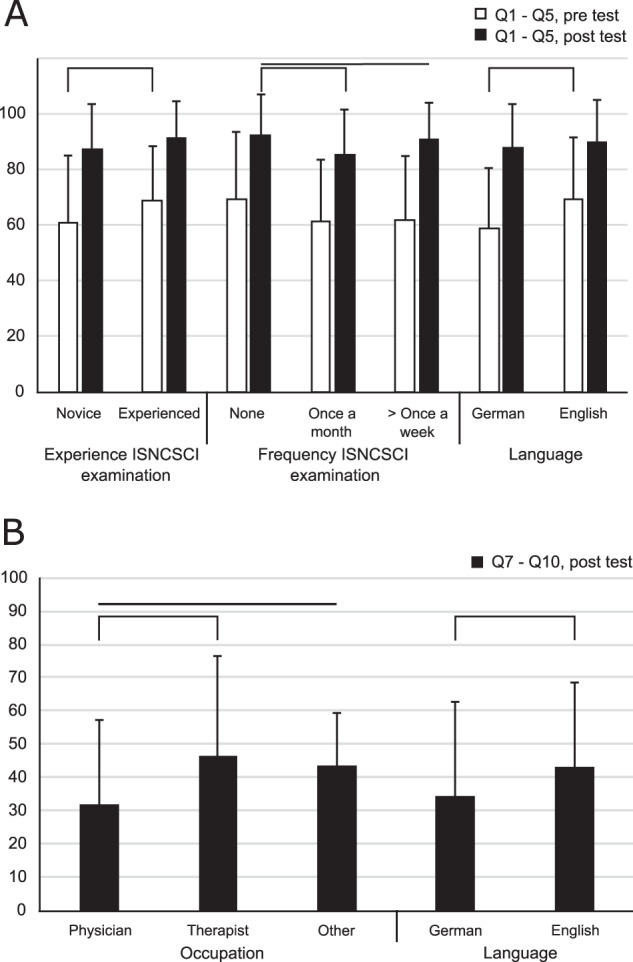
Relevant factors affecting the test results. Comparison of both significant differences in pre- and post-course test results for Q1–Q5 (**A**) and significant (sub-) group differences in test results for Q7–Q10 (**B**). On the *x*-axis, results are related to the relevant influencing factors (experience in ISNCSCI, frequency of ISNCSCI examination; **A**), relevant subgroups of participants (occupation; **B**), and language of the course (**A**, **B**). All results are illustrated as percentage of correctly keyed answers (*y*-axis). For a detailed record of all results please see Supplementary Material 3. Horizontal braces denote significant differences.

Two attendee-related factors were found, which have potential influence on the performance in Q1–Q5 either before or after the course: (1) the experience period in ISNCSCI examinations (*p*[pre] = 0.05, *p*[post] = 0.10; Fig. 3A), and (2) the frequency of regularly performing ISNCSCI examinations (*p*[pre] = 0.28, *p*[post] = 0.01; Fig. 3A). Accordingly, attendees with longer ISNCSCI experience performed better in the tests at the beginning of the courses as compared to novices (mean percentage of correct answers 69 ± 22% versus 61 ± 22%, *p* < 0.05). After the courses, no differences were found regarding the performance of these subgroups. A difference in respect to the frequency of performing ISNCSCI was only found in the post-course test (*p* = 0.01; Fig. 3A). Attendees performing ISNCSCI more frequently (“>once a week”: 91 ± 15%) seemed to achieve better results than those attendees practicing the ISNCSCI exam only once a month (“once a month”: 85 ± 15%). However, this difference was not significant (*p* > 0.05; Fig. 3A). Attendees who do not practice any ISNCSCI formally achieved better results than the two groups mentioned before (“none”: 91 ± 15%; *p* < 0.05 in relation to both “>once a week” and “once a month”; Supplementary Material 3).

The level of knowledge as measured by the mean percentage of correct answers in Q1–Q5 was independent from the profession of the attendees (*p*[pre] = 0.71, *p*[post] = 0.54; Supplementary Material 3).

The only difference in the answers to Q7–10 was a significantly superior performance of therapists as compared to physicians (46 ± 30% versus 32 ± 26%, *p* = 0.02; Fig. 3B).

### Course concept-related analysis

English-speaking course attendees showed superior pre-course test results in Q1–Q5 as compared to attendees of German-speaking courses (69 ± 21% versus 58 ± 23% for Q1–Q5, *p* < 0.01; Fig. 3A). These differences leveled off in the post-course test (89 ± 13% versus 88 ± 17%, *p* = 0.99; Supplementary Material 3). The course language has an impact on the results of the difficult, posttest-only questions Q7–Q10. Here, attendees of English-speaking courses achieved better results than those of the courses in German language (43 ± 26% versus 34 ± 29%, *p* = 0.03; Fig. 3B). The time concept of the course, meaning whether it was held in 1 or 1.5 days, affected the test results neither for Q1–Q5 (pre- and post-course: *p* = 0.61 and 0.37) nor for Q7–Q10 (solely post-course: *p* = 0.44; Supplementary Material 3).

### Question-related analysis including revisions

Q1–Q5, which included many crucial aspects of the ISNCSCI examination and were tested before and after each course, showed an overall improvement among all course participants (*p* < 0.01, McNemar chi-squared test, Table 2 and Fig. 1), confirming a higher level of knowledge in the examination guidelines (Table 2). The most difficult questions, as measured by the correct response rate in the posttest, were found to be Q5 (70%; “ISNCSCI examination position,” Fig. 1), Q2 (86%; “correct grading of light touch,” Fig. 1), followed by Q1 (88%; “correct grading pinprick,” Fig. 1). In contrast, both Q3 and Q4 achieved a correct response rate of more than 95% (Fig. 1).

**Table 2:** Post-course test results of questions 1–5 (Q1–Q5), grouped by the correctness of the answer in the pretest.

	Post-course test									
	Q1		Q2		Q3		Q4		Q5	
	×	✓	×	✓	×	✓	×	✓	×	✓
Pre-course test										
×	13	73	9	33	4	38	4	24	33	42
✓	5	64	9	83	0	114	2	126	10	59
McNemar statistic	57.6		12.6		36.0		17.0		18.5	
*p* value	<0.01		<0.01		<0.01		<0.01		<0.01	

Chi-square sampling distribution; degree of freedom = 1; the null hypothesis was rejected at a 0.05 significance level, leading to a critical value for the McNemar statistic of 3.84.

*Q* question, × incorrectly answered, ✓ correctly answered.

Of Q7–Q10, two questions were substantially revised during the study (Fig. 2, Q7A, B and Q9A, B, and Table 1): in their first version, these questions were correctly answered by only 21% (Fig. 2, Q7A) and 22% (Fig. 2, Q9A) of the course participants. The number of correct answers of Q7 did not even reach the simple majority. After a more precise phrasing, Q7 was correctly answered by 49% (Fig. 2, Q7B) and Q9 by 30% (Fig. 2, Q9B) of the attendees. Furthermore, the 40% of correct multiple responses in Q7 then represented the simple majority with respect to all chosen combinations of responses. The unmodified questions reached 52% (Q8, Fig. 2) and 42% (Q10, Fig. 2) of correct responses, also representing the most frequently chosen combination of answers.

## Discussion

Our results show that comprehensive ISNCSCI instructional courses do not only lead to improved scoring, scaling, and classification skills [9], but also enhance knowledge of the ISNCSCI examination guidelines and the underlying science. This is an important finding, because ISNCSCI is considered to be the gold standard to characterize the extent and level of the SCI [3] and competent examiners are a prerequisite for reliable and valid assessment. Even though practical skills are explicitly trained in EMSCI’s ISNCSCI instructional courses, testing knowledge of the guidelines does not necessarily equate to competency in exam performance. In order to resolve this discrepancy, practical exams would have to be implemented in the future (e.g., as “objective structured clinical examination—OSCE”) [17]. However, in the framework of a 1.5-day course, this is very challenging due to limited availability of assessors. Notwithstanding this issue, the combination of both theoretical lectures and practical hands-on bedside sessions seems to be very effective in knowledge transfer regarding the examination guidelines of ISNCSCI. All participants improved their knowledge regardless of profession or occupation. Even attendees improved, who were not routinely involved in ISNCSCI examinations or had not much experience in the field of SCI (Supplementary Material 3). In EMSCI, it is common practice that besides physicians, other medical professions including therapists and study nurses perform the ISNCSCI examination at any level of experience irrespective of a primarily clinical or scientific rationale (Supplementary Material 2). Even though attendees with higher experience in performing the ISNCSCI exam had a head start at the beginning of the course, the test results show that this advantage was no longer detectable at the end of the course (Fig. 3A). Completion of the course helped self-rated novices to achieve a steep learning curve. Finally, all of the groups from novices to experts gained a comparable knowledge level. According to our self-defined threshold the course was a success because the targeted 90% of correctly answered questions in the posttest was achieved by the majority of the participants (Fig. 1). In this connection, it must be noted that exceeding the 90% correctness rate already means a de facto error-free result (100% correct answers), considering that five questions were included in the pre–post evaluation. In consequence, more than half of the attendees achieved an immaculate posttest result. In contrast, a higher frequency of regular execution of the ISNCSCI examination did not lead to better results at the beginning of the course (Fig. 3A). Among those participants who conduct ISNCSCI exams more frequently (>once a week) than others (once a month), this did not change to any relevant extent even at the end of the course (Fig. 3A). Surprisingly, attendees who had never conducted any ISNCSCI examinations revealed a better performance according to the post-course test results (Fig. 3A). However, we consider the significant differences of <1 and 6% as not relevant in the light of a > 90% correct response rate. Thus, underlying reasons remain unclear at this point.

In the posttest, the results of the explicitly demanding questions Q7–Q10 show a significantly superior performance of therapists compared to physicians. A likely explanation is the content of these questions referring mainly to details of the motor examination, which, within the EMSCI network, is performed by therapists rather than physicians.

Our results are in line with another publication confirming successful learning effects after 2 h of self-study using the official ISNCSCI booklet [18]. This study by Liu et al. [10] used a ten-item questionnaire as success control in 46 medical students. While our study focuses exclusively on testing knowledge of the examination guidelines, the questionnaire of Liu et al. also involved classification guidelines and was only based on polar questions (true versus false statement).

### Principles of the course concept

The course language represented the only finding related to the course concept that influenced the results of both parts of the success monitoring, the pre- (Q1–Q5) and the post-course test (Q7–Q10). Accordingly, attendees of English-speaking courses showed better test results in Q1–Q5 already before the course and achieved a lower error rate in Q7–Q10 after the course, as compared to those of German-speaking courses (Fig. 3A, B). Whether factors like the use of English slides in the theoretical part of both the German and the English courses might have affected these results has yet to be determined. Most important, all attendees in both course types (English and German) reached a comparable level of test performance at the end of the course (Fig. 3A).

For organizational reasons, three of the courses were organized at locations other than Heidelberg. These courses were held over 1 day. Overall, the test results of attendees were comparable in both the 1- and 1.5-day courses (Supplementary Appendix 3). However, attendees’ testimonials suggest time pressure and information overload, possibly leading to inferior long-term/carry-over effects [19, 20]. Long-term effects were not evaluated in this study, though.

### General considerations regarding the success monitoring

Five questions (Q1–Q5) were tested in a pre- and post-course approach, enabling to evaluate short-term improvements in ISNCSCI examination-related knowledge. This led to the proof of an equal level of knowledge of all attendees after having completed the course. In addition, four questions (Q7–Q10) were only tested at the end of the course to improve selectivity between good and excellent attendees. To achieve this, a considerably challenging content for novices was chosen. Furthermore, three of the four items of Q7–Q10 were of the type “multiple response,” which is considered to be particularly challenging [14]. Thus, a response rate of 36 ± 14% correct answers in Q7–Q10 versus 89 ± 11% in Q1–Q5 might give a confirmative hint that mainly excellent attendees have answered these questions correctly (Figs. 1 and 2).

### Specific considerations regarding the success monitoring and its contents

Besides Q5, which will separately be discussed below, Q1 and Q2 appear to be particularly conspicuous (Fig. 1). The correct assessment of pinprick, which is the content of Q1 (Table 1), and the grading of light touch, which again is the content of Q2 (Table 1), proved to be the most difficult tasks among those that were tested before and after the course (Table 2).

This finding is remarkable since the pinprick appreciation (Q1), rather than the light touch examination (Q2), is frequently considered to be the most challenging part of the sensory examination of the ISNCSCI, particularly when considering the general psychometric properties and assessing incomplete lesions [21]. Apparently, the rule that an evoked paresthesia already counts as altered perception (grade of 1) in both light touch sensation and pinprick appreciation seems to be an exceptional challenge (Figs. 1, Q1, and 2). Therefore, we recommend emphasizing both the role of altered sensation and the differences between a grade of 1 in light touch and pinprick in the ISNCSCI training.

Q5 was the only question of Q1–Q5 that represented the type “multiple response,” which is considered to be exceptionally difficult [14]. In addition, item (c) of Q5 describes the crucial rule regarding the required supine position of the patient. This item was correctly keyed by 96% of the attendees (*N* = 143; Fig. 1), if evaluated isolated. In comparison, item (a) of Q5 was focused on the potential lateral positioning of the patient for the anorectal examination. From a clinical point of view, however, this aspect is deemed less relevant compared to the basic rule of the supine position. This certainly implies room for improvement regarding the structure of Q5 when considering future revisions of the questionnaire.

Q7 and Q8 tested the understanding of the most crucial part in the ISNCSCI exam, the anorectal examination. The results of Q8 (nearly 52% completely correct and additional 34% partially correct responses) suggest that the participants indeed understood how to check for “Deep Anal Pressure” sensation (Fig. 2, Q8). However, more than 46% of the attendees were not able to reliably recall how to avoid false positive findings in the motor examination of the external anal sphincter muscle (Q7) due to reflex contractions, e.g., triggered by a Valsalva maneuver (Fig. 2, Q7). But still, almost three-fourths of the participants were able to provide correct answers to the question of how to technically perform this examination. These results underline that it is very important to carefully and explicitly impart all the details of the ISNCSCI examination guidelines in order to achieve valid examination results.

Finally, when compiling a questionnaire for success monitoring, it should be considered that ISNCSCI instructional courses primarily aim to ensure that a minimum standard of quality is achieved. Therefore, also supposedly simple questions, like for instance Q3 and Q4 (Table 1 and Fig. 1), should be included in order to make sure that basic knowledge of ISNCSCI, which is indispensable for novices, was satisfactorily imparted [22].

### Study limitations

As already stated within the discussion, the main limitation of the study is that albeit targeting to evaluate knowledge of guidelines regarding the practical examination according to ISNCSCI, in fact no practical skills were tested using the presented written pre–posttest concept. However, it was attempted to shape the questions of the test as practically oriented as possible. A further limitation of the study concerns its pre–post design. Thus, a bias could have occurred if the participants had reviewed/checked their responses to the questionnaire based on their memory in the time between the two tests. However, the participants were neither informed about the correct items of the questionnaire after the pretest nor that the same questions were posed in the posttest. Moreover, it might even have been favorable if the participants had been self-motivated to enlarge their knowledge upon their experiences in the pretest. Notwithstanding this, the extent of both such a bias and long-term learning effects could have been evaluated by means of a delayed retest or a control group of individuals, who would have been asked to complete the same questionnaire without having attended a hands-on training course. The recruitment of a control group with matched profession and experience represents a highly challenging task, though. Finally, it might be assumed that remaining gaps in knowledge, such as present here, could compromise the accuracy and reliability of the ISNCSCI exam results needed in clinical trials. However, achieving a 100% correctness rate in posttests after a 1.5-day course seems highly unlikely, in particular in novice examiners. Rather repeated refresher courses and constant practical application of the ISNCSCI could help to optimize the reliability and knowledge of the standards.

## Conclusion and future perspectives

Combined theoretical and hands-on ISNCSCI training is strongly recommended to ensure a high level of knowledge in the ISNCSCI examination guideline in clinical routine as well as research, reflected by a substantial gain of knowledge of the participants. This is of importance since instructional courses are capable of conveying knowledge and practical skills, which cannot be performed and considered by calculators [23], such as the recognition of neurological impairments that are not related to SCI [8, 24]. Aside from learning particular demanding aspects of ISNCSCI, the effectiveness of such courses does not depend on the profession and or the experience in ISNCSCI and spinal cord medicine. Based on our results, there is no need for courses specifically focusing on attendees’ different grades of experience or profession. Success monitoring by means of an elaborated questionnaire covering the most important rules of the examination is recommended. The implemented questionnaire only evaluated the immediate effects directly after the instructional course. In future courses, it should be considered to reevaluate the knowledge after a reasonable period of time. We hypothesize that the frequency of conducting ISNCSCI exams is associated with a better performance in such “mid-term” retests. Prospectively, it certainly appears reasonable to also evaluate the expected benefit of the already implemented hands-on training by means of practical exams, such as the OSCE.

In principle, internationally harmonized instructional courses on the ISNCSCI examination and classification guidelines might represent a valuable approach to yield broadly approved and equally trained ISNCSCI assessors. This is of importance, not only to meet both the demands of existing SCI guidelines and the “International Council for Harmonization of Technical Requirements for Pharmaceuticals for Human Use guideline for good clinical practice,” but also to facilitate international collaborations [25–28].

## Supplementary information

Supplemental Material 1-3

## Data Availability

The data sets generated and/or analyzed during the current study are available from the corresponding author on reasonable request.
