# Supplementary material for: Theoretical and practical training improves knowledge of the examination guidelines of the International Standards for Neurological Classification of Spinal Cord Injury
Source: Spinal Cord. 2020 Nov 17;60(1):1–10. doi: 10.1038/s41393-020-00578-1 (PMC8737333; doi:10.1038/s41393-020-00578-1)
Supplement: Supplementary file 1 — Supplemental Material 1-3 [file 41393_2020_578_MOESM1_ESM.pdf]

S. Franz, L. Heutehaus, S. Weinand, N. Weidner, R. Rupp, and C. Schuld, Theoretical and practical training improves knowledge of the examination guidelines of the International Standards for Neurological Classification of Spinal Cord Injury, *Spinal Cord*, 2020.

Supplementary Material 1 Participants' questionnaire for the ISNCSCI instructional course

European Multicenter Study about Spinal Cord Injury (EMSCI)	**Participants questionnaire ISNCSCI Instructional Course**	SCI Center Heidelberg University Hospital

Name or pseudonym: ____

Occupation:
- ☐ physician
  - ☐ neurologist (registrar or consultant)
  - ☐ orthopaedic specialist (registrar or consultant)
  - ☐ specialist in rehabilitation and physical medicine (registrar or consultant)
- ☐ physical therapist
- ☐ occupational therapist
- ☐ nurse
- ☐ other rehabilitation professional: ____

Experience in spinal cord medicine:
- ☐ - 1 year
- ☐ 1 year to 5 years
- ☐ 6 years to 10 years
- ☐ more than 10 years

Experience in the **examination** part of the ISNCSCI protocol:
- ☐ expert
- ☐ highly experienced
- ☐ experienced
- ☐ novice

Experience in the **scoring, scaling and classification** part of the ISNCSCI protocol: (How to determine the ASIA Impairment Scale, the Neurological Level of Injury etc.)
- ☐ expert
- ☐ highly experienced
- ☐ experienced
- ☐ novice

How often do you perform ISNCSCI examinations?
- ☐ once a day
- ☐ twice a week
- ☐ once a week
- ☐ once a month
- ☐ never or less often than once a month

S. Franz, L. Heutehaus, S. Weinand, N. Weidner, R. Rupp, and C. Schuld, Theoretical and practical training improves knowledge of the examination guidelines of the International Standards for Neurological Classification of Spinal Cord Injury, *Spinal Cord*, 2020.

Supplementary Material 1 Participants' questionnaire for the ISNCSCI instructional course

European Multicenter Study about Spinal Cord Injury (EMSCI)	Participants questionnaire ISNCSCI Instructional Course	SCI Center Heidelberg University Hospital

In following part, we would like to assess some questions regarding ISNCSCI's examination part. All questions with 5 choices (a-e) have only one correct answer (single choice). All questions with 4 choices (a-d) have one or more correct answers (multiple choice), which mean that all single choices have to be evaluated for correctness.

1) A patient reports a strong tingling sensation (German: kribbeln) when being tested with both ends of the safety pin in the pin prick examination. Due to this sensation the patient cannot reliably distinguish between the sharp and dull end of the safety pin. What is the correct grading? (single choice)
   (a) Grade 0
   (b) Grade 1
   (c) Grade 2
   (d) Not testable
   (e) Grade 1 with an additional note in the comment box

2) The patient reports the same tingling sensation when being touched with the cotton tip in the light touch examination. What is the correct grading? (single choice)
   (a) Grade 0
   (b) Grade 1
   (c) Grade 2
   (d) Not testable
   (e) Grade 1 with an additional note in the comment box

3) Which tool does the ASIA define for testing pin prick discrimination? (single choice)
   (a) Pointed fingernails and fingertips
   (b) Both ends of an injection needle
   (c) Both ends of a stretched apart disposable safety pin
   (d) Broken wooden pick and cotton tip
   (e) Both sides of a thumbtack (German: Reißzwecke)

Supplementary Material 1 Participants' questionnaire for the ISNCSCI instructional course

4) What is the meaning of grade 3 in the motor examination? (single choice)
   (a) Active movement, directed laterally away from center of body
   (b) Active movement, full range of motion against gravity
   (c) Active movement, full range of motion with gravity eliminated
   (d) Active movement, directed towards the center of body
   (e) None of the above

5) In which position has the patient to be for the ISNCSCI examination? (multiple choice)
   (a) Lateral position for the anorectal examination (German: Seitenlage)
   (b) The upper extremities in lateral position, the lower extremities in supine position
   (c) Supine position (German: Rückenlage)
   (d) In the wheelchair

6) How many key muscles are tested per body side in ISNCSCI's motor examination? (single choice)
   (a) 9
   (b) 10
   (c) 11
   (d) 12
   (e) 20

7) **A:** While testing voluntary anal contraction according to ISNCSCI, you, as the examiner, feel a contraction. Which of the following procedures can help in distinguishing a voluntary from a reflex anal contraction? (multiple choice)
   (a) Due to SCI, an involuntary contraction is not expected
   (b) I ask the patient to release my finger, while I feel the contraction
   (c) I ask the patient for a Valsalva maneuver
   (d) I confirm my findings by electromyography of the external sphincter

7) **B:** While testing voluntary anal contraction according to ISNCSCI, you, as the examiner, feel a contraction. Which of the following procedures can help in distinguishing a voluntary from a reflex anal contraction? (multiple choice)
   (a) Due to SCI, an involuntary contraction is not expected
   (b) I ask the patient to release my finger, while I feel the contraction
   (c) If the contraction can only be elicited by a Valsalva maneuver, a pure reflex contraction is most probable explanation
   (d) I confirm my findings by electromyography of the external sphincter

8) ISNCSCI also contains an examination for deep anal pressure to evaluate the sensory fibers of the spinal segments S4-5. How is it tested? (multiple choice)
   (a) Insert the tip of your index finger and squeeze the skin of the external sphincter with your thumb against your index finger
   (b) Apply gentle pressure to the anorectal wall (innervated by the somatosensory components of the pudental nerve S4-5)
   (c) You have to assure that the complete finger is inserted
   (d) It is tested for both body sides

9) **A:** The C6 key muscle examination (M. ext. carpi radialis) for grades 4, 5 requires the examiner to put resistance against the patient's movement. To which patient movement do you as the examiner direct the resistance? (multiple choice)
   (a) Extension
   (b) Abduction of the hand towards ulna in frontal plane
   (c) Flexion
   (d) Abduction of the hand towards radius in frontal plane

9) **B:** The C6 key muscle examination (M. ext. carpi radialis) for grades 4, 5 requires the examiner to put resistance against the patient's movement. To which direction do you as the examiner put the resistance? (single choice)
   (a) Extension
   (b) Abduction of the hand towards ulna in frontal plane with flexion
   (c) Flexion
   (d) Abduction of the hand towards radius in frontal plane
   (e) Abduction of the hand towards radius in frontal plane and Extension

10) A common compensatory movement during the S1 (plantar flexion) examination in the grade 3 position is the following active movement? (single choice)
   (a) Ankle dorsiflexion
   (b) Knee extension
   (c) Hip flexion
   (d) Hip extension
   (e) None of the above

S. Franz, L. Heutehaus, S. Weinand, N. Weidner, R. Rupp, and C. Schuld, Theoretical and practical training improves knowledge of the examination guidelines of the International Standards for Neurological Classification of Spinal Cord Injury, *Spinal Cord*, 2020.

Supplementary Material 2 Detailed characterization of course participants

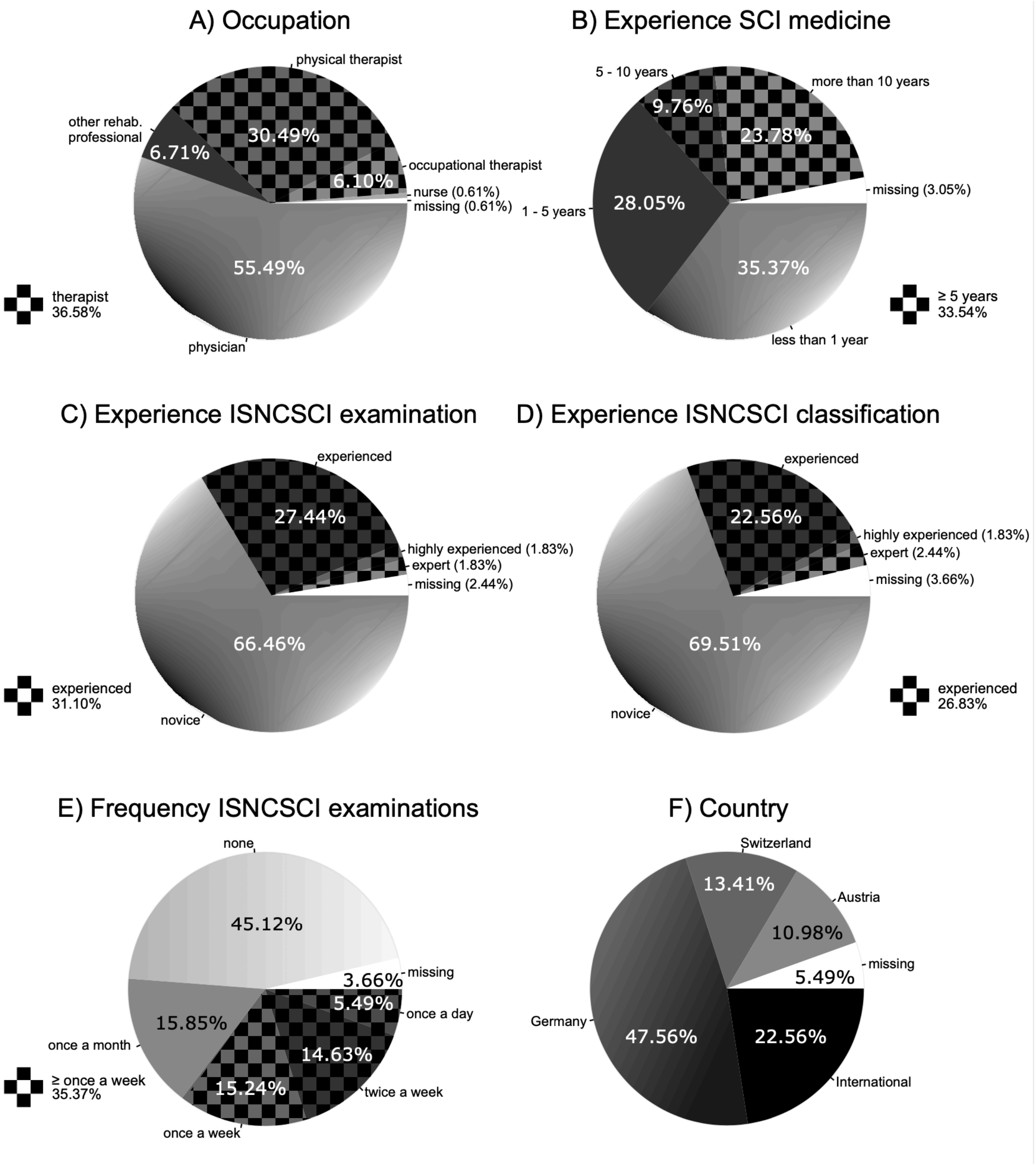

Checkered sectors within pie-charts represent pooled subgroups of self-rated (individual) characteristics of course attendees.

S. Franz, L. Heutehaus, S. Weinand, N. Weidner, R. Rupp, and C. Schuld, Theoretical and practical training improves knowledge of the examination guidelines of the International Standards for Neurological Classification of Spinal Cord Injury, *Spinal Cord*, 2020.

Supplementary Material 3 Nonparametric analyses of the amount of correctly keyed pre/post-course questions dependent on influencing factors

**Factor**	**Q1-Q6, pre-test**				**Q1-Q5, post-test**				**Q7-Q10, post-test**			
**Occupation**	N	mean	SD	p values	N	mean	SD	p values	N	mean	SD	p values
Physician [1]	86	61.6	24.3	0.708	86	87.3	16.2	0.541	89	31.7	25.5	0.001
Therapist [2]	60	65.4	19.8		60	89.9	13.1		60	46.2	30.1	[1] - [2] 0.015
Other [3]	10	63.5	24.3		10	87.0	14.8		11	43.2	16.2	
**Experience SCI medicine**												
<= 1 year [1]	57	59.6	22.4	0.185	57	87.6	16.4	0.408	58	31.5	23.7	0.359
1-5 years [2]	45	63.6	23.3		45	90.9	13.4		45	43.9	31.6	
> 5 years [3]	54	66.6	22.1		54	86.9	15.6		54	41.2	27.1	
**Experience ISNCSCI examination**												
novice [1]	107	60.6	22.3	0.046	107	87.0	15.6	0.099	108	37.3	28.5	0.359
experienced [2]	49	68.7	22.5		49	91.1	14.4		49	40.8	25.9	
**Experience ISNCSCI classification**												
novice [1]	111	62.4	21.5	0.441	111	88.3	15.2	0.930	112	40.2	28.5	0.344
experienced [2]	43	64.8	25.7		43	88.2	15.9		43	34.9	25.1	
**Frequency ISNCSCI examination**												
> once a week [1]	56	63.1	22.8	0.277	56	90.9	15.2	0.012	56	33.5	24.4	0.234
once a month [2]	72	61.0	21.8		72	85.1	15.1	[1] - [3] 0.005	26	44.2	28.6	
none [3]	26	68.7	24.9		26	91.5	15.2	[2] - [3] 0.011	73	40.8	29.3	
**Language**												
German	89	58.5	23.0	0.004	89	87.5	17.1	0.998	92	34.0	28.6	0.030
English	67	69.3	20.6		67	89.4	12.7		69	42.8	25.8	
**Duration**												
1 day	32	66.2	21.8	0.611	32	86.2	15.6	0.365	33	42.4	31.6	0.444
1.5 days	124	62.4	22.8		124	88.8	15.3		128	36.5	26.6	

The different factors (1st column) are related to the self-rating of participants stated in the pre-test-questionnaire. Values in square brackets mark subgroups of given factors. Unattached p values denote significance of differences between given factors. According to the post-hoc testing, p values attached to values in square brackets denote significance between the stated subgroups. **Abbreviations:** question number **Q**; sample size **N**; mean percentage of correct answers **mean**; standard deviation **SD**.
